# Europhysiology 2022: bringing together the physiologists of Europe and the World: 16–18 September 2022, Tivoli Congress Center, Copenhagen, Denmark” as subtitle

**DOI:** 10.1007/s00424-022-02676-w

**Published:** 2022-03-07

**Authors:** Ralf P. Brandes, Stefan Gründer

**Affiliations:** 1 Goethe-University, Frankfurt, Germany; 2 RWTH Aachen University, Aachen, Germany

Physiology is not only the most central aspect of biomedical life sciences, but also one of its largest areas. Being the “science of life” means that virtually every research concerning cells and animals also has a twist towards physiology. This makes this discipline so important that the Nobel Prize is awarded to “Medicine and Physiology”. In times of exponential growth of scientific knowledge and — owing to this — a massive diversification of research, smaller physiological meetings without a precise thematic focus are struggling. The diversity of the audience limits the number of qualified interaction partners. As a consequence, there has been a tendency towards highly specialised scientific meetings.

The Europhysiology meeting series is the answer of Europe’s physiologists to this development. A pan-European meeting with an audience far beyond 1000 attendees and the reputation to attract the most famous speakers offers a key audience for each speciality and cutting-edge science at the highest level. The Europhysiology meeting series started in 2018 with a successful and very well received first meeting in London. Due to COVID-19, the 2020 meeting planned for Berlin had to be cancelled, but the meeting series will continue as agreed by the hosting societies.

In this year, 2022, another Europhysiology meeting will take place. Europhysiology 2022 will be an in-person event at the Tivoli Garden’s conference center (https://www.tivoli.dk) in the beautiful city of Copenhagen from September 16 to 18, 2022.

There are many reasons to attend this meeting.

- The programme is scientifically exciting and well balanced. The four hosting societies, The Physiological Society, The Scandinavian Physiological Society, The German Physiological Society, and the Federation of European Physiological Societies, have developed a programme that not only is diverse, but also showcases the most recent developments in science and a breadth of exciting topics. More information can be found at https://europhysiology2022.org/programme-16-sept/.
- The conference offers various opportunities for informal contact, scientific exchange, and networking. We all have missed the direct interaction with our peers during the COVID-19 pandemic. Finally, this option becomes available again with Europhysiology!
- Europhysiology offers numerous possibilities for young researchers to increase their visibility. From a pre-symposium of the “Young Physiologists”, to sessions on career development and numerous awards. Poster and oral sessions shaped from selected abstracts offer ample possibilities for emerging researchers to present their work.
- Focussed symposia in the main congress programme as well as pre-conference meetings of special interest groups in almost all areas of physiology assure that also more focused interactions are possible.
- Finally, and aside from science, the cosmopolitan capital of Denmark, *København*, never disappoints. It is a city not only easy to reach but also full of vibrant culture, good food, lasting impressions, and exciting history!

Abstract to the meeting can be submitted under https://europhysiology2022.org/ until May 25th.

We look forward to welcoming you all, whatever your career stage, in Copenhagen at Europhysiology 2022. Let’s meet for real!

